# The meshless local Petrov–Galerkin method based on moving Kriging interpolation for solving the time fractional Navier–Stokes equations

**DOI:** 10.1186/s40064-016-2047-2

**Published:** 2016-04-06

**Authors:** N. Thamareerat, A. Luadsong, N. Aschariyaphotha

**Affiliations:** Department of Mathematics, Faculty of Science, King Mongkut’s University of Technology Thonburi (KMUTT), 126 Pracha Uthit Rd., Bang Mod, Thung Khru, Bangkok 10140 Thailand; Ratchaburi Learning Park, King Mongkut’s University of Technology Thonburi (KMUTT), Rang Bua, Chom Bueng, Ratchaburi 70150 Thailand; Theoretical and Computational Science Center (TaCS), Science Laboratory Building, Faculty of Science, King Mongkut’s University of Technology Thonburi (KMUTT), 126 Pracha Uthit Rd., Bang Mod, Thung Khru, Bangkok 10140 Thailand

**Keywords:** Meshless local Petrov–Galerkin method, Moving Kriging interpolation, Quadrature formula, Time fractional Navier–Stokes equations

## Abstract

In this paper, we present a numerical scheme used to solve the nonlinear time fractional Navier–Stokes equations in two dimensions. We first employ the meshless local Petrov–Galerkin (MLPG) method based on a local weak formulation to form the system of discretized equations and then we will approximate the time fractional derivative interpreted in the sense of Caputo by a simple quadrature formula. The moving Kriging interpolation which possesses the Kronecker delta property is applied to construct shape functions. This research aims to extend and develop further the applicability of the truly MLPG method to the generalized incompressible Navier–Stokes equations. Two numerical examples are provided to illustrate the accuracy and efficiency of the proposed algorithm. Very good agreement between the numerically and analytically computed solutions can be observed in the verification. The present MLPG method has proved its efficiency and reliability for solving the two-dimensional time fractional Navier–Stokes equations arising in fluid dynamics as well as several other problems in science and engineering.

## Background

In recent years, substantially contributed works associated with the fractional differential equations (FDE), also sometimes called as extraordinary differential equations, have been published both theoretical and numerical aspects on account of its applications in almost all branches of sciences and engineering. Various physical phenomena in fluid mechanics, viscoelasticity, control theory of dynamical systems, chemical physics, biology, stochastic processes, finance, and other sciences can be successfully described by fractional models. Even though there have been a lot of achievements on the theoretical analysis, exact solutions of most FDE cannot be derived explicitly. Only approximate analytical and numerical solutions can be obtained using procedures such as linearization, perturbation, or discretization. As a result, proposing a new algorithm to find the numerical solution of FDE is of practical significance. There are several definitions to the generalization of the notion of differentiation to fractional orders, including the Grünwald–Letnikov’s definition, Riemann–Liouville’s definition, Caputo’s definition, Jumarie’s definition, Chen’s definition, and local fractional (fractal) Yang derivative. Each of these fractional derivatives presents both advantages and disadvantages. The two most frequently used generalizations of the derivative are the Riemann–Liouville’s and Caputo’s definition. The Riemann–Liouville fractional derivative is not suitable to deal with the physical problems in the real world since it requires the definition of fractional order initial conditions, which have no physically meaningful interpretation yet. Caputo introduced an alternative definition in which the initial conditions for the fractional order differential equations can be given in the same manner as for the integer order differential equations with a known physical interpretation. Another main advantage of the Caputo fractional derivative over the definition of Riemann–Liouville is that the Caputo derivative of a constant function is zero whereas the same does not hold for the Riemann–Liouville derivative. It is reasonable that the fractional derivative of a constant should be zero from a physical point of view. We refer the interested reader to the review article of De Oliveira and Tenreiro Machado (2014) in order to learn more details about a brief overview of proposed definitions of fractional integrals and derivatives.

The classical Navier–Stokes equations (NSE), developed by Claude-Louis Navier and George Gabriel Stokes in 1822, are the fundamental equations of fluid dynamics that extensively studied both theoretically and numerically. Many numerical researches have reported that the NSE are reasonably suitable for describing the mathematical modeling and numerical simulation relating to the flow behavior of fluids. Mathematically, the NSE are a time dependent system of nonlinear second-order partial differential equations (PDE) which can be derived from the basic conservation laws for mass and momentum. The time fractional NSE having been recently proposed by El-Shahed and Salem (2004) can be obtained by simply replacing the first-order time derivative term by derivative of non-integer order but retaining the first- and second-order space derivatives. To obtain the solution of the time fractional NSE has never been an easy task due to the nonlinearity which makes them quite difficult or impossible to solve. There are very few cases in which the solution of fractional NSE can be solved analytically. We have to make certain simplifications and assumptions about the state of fluid and configuration for the pattern of flow is to be considered. Several analytical methods have been proposed and developed for the solution of the time-fractional NSE. El-Shahed and Salem (2004) employed the Laplace, Fourier sine and finite Hankel transforms to obtain the exact solution for three different special cases of the time fractional NSE including the flow in a long circular pipe, the flow due to a plane wall suddenly set in motion, and the flow in plane Coutte motion. Momani and Odibat (2006) constructed the explicit solutions of the time-fractional NSE in cylindrical coordinates for an unsteady one-dimensional motion of a viscous fluid by Adomian decomposition method (ADM). In this scheme, the analytic solution is formed in terms of series with easily computable term. The effective Adomian’s method was extended to determine the analytical approximate solution of the two-dimensional time fractional NSE in Cartesian coordinates by Wang et al. (2009). The pressure gradient term was also assumed to be a constant which make the fractional model easy to implement. Shortly after that, the investigation with regard to solving the one-dimensional time fractional NSE in cylindrical coordinates has emerged continuously. Ragab et al. (2012) employed the homotopy analysis method (HAM) to obtain an approximate solution of the NSE with fractional order in the form of power series. They also pointed out that both the homotopy perturbation method (HPM), ADM and variational iteration method (VIM) are special cases of the HAM. Kumar et al. (2014) introduced a new analytical and approximate technique based on ADM and Laplace transform method (LTM) to obtain the solution of time-fractional NSE of a viscous fluid in a tube. Kumar et al. (2015) presented a reliable approach based on the new homotopy perturbation transform method (HPTM) to seek the analytical solution of NSE with time fractional derivative in a tube. Almost all the accomplishments on the theoretical analysis to acquire the solution of such a problem in the literature are only limited to one dimension. None of the above schemes is completely satisfactory when dealing with the multi-dimensional problems of the time fractional NSE. To fill this gap, particular attention in this work is devoted to the development of computational techniques and numerical strategies to the primitive variables formulation (velocity and pressure) of the time fractional NSE in two dimensions.

On the other hand, so far, only few researchers have attempted to extend and develop the so-called meshless methods to the solution of the time fractional NSE. The meshless or meshfree methods are the recently developed numerical technique which can be alternatively used to overcome the difficulties and limitations of mesh generation. Academic works regarding the applications of meshless methods have received considerable interest and have been published both theoretical and numerical aspects (Buhmann 2003; Kaufmann et al. 2010, 2012; Li et al. 2010; Abbasbandy et al. 2012, 2014; Shirzadi et al. 2012; Ala and Francomano 2013, 2015; Dai et al. 2013a, b; Yimnak and Luadsong 2014; Khankham et al. 2015; Phaochoo et al. 2016a, b; Tu et al. 2016). The meshless methods are used to establish the system of algebraic equations for the whole problem domain without the use of a predefined mesh or domain discretization. Generally speaking, in accordance with the formulation procedure, meshfree methods fall into three categories: meshfree weak form methods, meshfree strong form methods, and meshfree weak–strong form methods. This work is particularly focused on the meshfree weak form methods. The common characteristic of weak form methods is that the original PDE of a problem is converted into an integral equation globally or locally based on a principle of weighted residual methods. There are a great number of meshfree methods that use local nodes for approximating the field variable, for example, the element free Galerkin (EFG) method (Belystchko et al. 1994), the meshless local Petrov–Galerkin (MLPG) method (Atluri and Zhu 1998a, b), the reproducing kernel particle method (RKPM) (Liu et al. 1995), the point interpolation method (PIM) (Liu and Gu 2001a), the radial point interpolation method (RPIM) (Liu and Gu 2001b; Wang and Liu 2000, 2002) and so forth. Some typical meshless methods based on global weak forms such as the EFG, RKPM, and RPIM, being “meshless” only in terms of the interpolation of the field variables, have to use background cells to evaluate integrals appearing in the local weak formulation. This is one reason why the aforementioned methods are not truly meshless method. One of the most popular meshless methods is the meshless local Petrov–Galerkin (MLPG) method which was first proposed by Atluri and Zhu (1998a, b) for solving linear potential problems. The MLPG approach is referred to as a one of the truly meshless methods which is used much more widely than other existing methods. In this method, a nodal sub-domain is used in place of background integration cells in order that all integrations are carried out locally over small sub-domains of regular shapes. In addition, the MLPG method is also different from the truly meshless method based on the local boundary integral equation (LBIE) method (Zhu et al. 1998a, b) in the fact that there is no singular integral in the present MLPG method, while some kinds of singular integrals have to be tackled in the meshless LBIE method (Atluri and Zhu 2000). The concept of shape function construction is one of the central and most important issues that significantly effect on the performance of meshfree methods. A number of ways to efficiently create shape functions have been proposed including the moving least squares (MLS) approximation, radial point interpolation method (RPIM), and moving Kriging (MK) interpolation. The MLS shape functions do not have the Kronecker delta property thereby making the imposition of essential boundary conditions complicated. Meanwhile, Dai et al. (2003) has proven that the Kriging interpolation is essentially the same as the RPIM as long as the same basis functions are used. That is properties found in RPIM should apply to Kriging shape function as well. In this study, we employ the moving Kriging (MK) interpolation technique, which was first introduced in computational mechanics by Gu (2003). One notable feature of shape function constructed using the MK interpolation is that it possesses the Kronecker delta property which satisfies the essential boundary conditions automatically. The essential boundary conditions can be easily implemented without any special treatments.

As mentioned previously, there still lacks research efforts that have utilized the MLPG method to investigate the time fractional model of NSE in Cartesian coordinates. To the best of our knowledge, such a task has not yet been carried out while this work is being reported. The objective of this work is to extend further the application of the MLPG method to the NSE of fractional order. We organize the rest of this paper as follows. “The moving Kriging interpolation method” section gives some basic concepts of the moving Kriging interpolation for constructing shape functions in meshfree procedure. In “Research methodology”, we introduce the governing two-dimensional time fractional NSE in Cartesian coordinate system and then we describe how to formulate the standard weak formulation and establish the discretized system equations. In “Numerical experiments and results” section, the numerical experiments are presented and discussed in detail to demonstrate and confirm the accuracy and efficiency of the proposed scheme. Finally, we complete the paper with some conclusion given in “Conclusion” section and some references are listed at the end.

## The moving Kriging interpolation method

Kriging or Gaussian process regression was originally applied in geostatistics for spatial interpolation. Subsequently, Kriging interpolation was employed to construct shape functions for enhancement of the meshfree methods, intimately related to generalization of finite element methods (FEM). The procedure of constructing shape functions for meshfree methods using the MK interpolation is detailed in this section. Let us consider a sub-domain, $\Omega _{s} \subset\Omega ,$ the neighborhood of a point **x** and denoted as the domain of definition of the MK interpolation for the trial function at **x**. To approximate a distribution function *u* in $\Omega _{s}$ over a number of randomly located nodes $\left\{ {{\mathbf{x}}_{i} ,i = 1,2,3, \ldots N} \right\}$ where *N* is the total number of nodes in the sub-domain, the formulation of the MK interpolation $u^{h} \left( {\mathbf{x}} \right),\forall {\mathbf{x}} \in\Omega _{s}$ can be expressed in the form of linear combination of the shape functions:

1 $$u^{h} \left( {\mathbf{x}} \right) = \mathop \sum \limits_{j = 1}^{N} \phi_{j} \left( {\mathbf{x}} \right)\hat{u}_{j} = {\varvec{\Phi}}\left( {\mathbf{x}} \right){\mathbf{U}}, \quad \forall {\mathbf{x}} \in\Omega _{s} ,$$

where ${\mathbf{U}} = \left[ {\hat{u}\left( {{\mathbf{x}}_{1} } \right) \hat{u}\left( {{\mathbf{x}}_{2} } \right) \hat{u}\left( {{\mathbf{x}}_{3} } \right) \ldots \hat{u}\left( {{\mathbf{x}}_{N} } \right)} \right]^{T}$ and ${\varvec{\Phi}}\left( {\mathbf{x}} \right)$ is a $1 \times N$ vector of Kriging shape function defined by

2 $${\varvec{\Phi}}\left( {\mathbf{x}} \right) = {\mathbf{p}}^{T} \left( {\mathbf{x}} \right){\mathbf{A}} + {\mathbf{r}}^{T} \left( {\mathbf{x}} \right){\mathbf{B}}.$$

The matrices **A** and **B** are determined by

3 $${\mathbf{A}} = \left( {{\mathbf{P}}^{T} {\mathbf{R}}^{ - 1} {\mathbf{P}}} \right)^{ - 1} {\mathbf{P}}^{T} {\mathbf{R}}^{ - 1} ,$$

4 $${\mathbf{B}} = {\mathbf{R}}^{ - 1} \left( {{\mathbf{I}} - {\mathbf{PA}}} \right),$$

where **I** is the unit matrix of size $N \times N$ and **p**(**x**) is a vector of the polynomial with *m* basis functions given by

5 $${\mathbf{p}}\left( {\mathbf{x}} \right) = \left[ {p_{1} \left( {\mathbf{x}} \right) p_{2} \left( {\mathbf{x}} \right) p_{3} \left( {\mathbf{x}} \right) \ldots \,p_{m} \left( {\mathbf{x}} \right)} \right]^{T} .$$

The commonly used linear basis in two-dimensional space is given by

$${\mathbf{p}}^{T} \left( x \right) = \left\{ {1,x,y} \right\},\,\,m = 3,$$

the quadratic polynomial basis is

$${\mathbf{p}}^{T} \left( x \right) = \left\{ {1,x,y,x^{2} ,xy,y^{2} } \right\},\,\,m = 6,$$

and the cubic polynomial basis is

$${\mathbf{p}}^{T} \left( x \right) = \left\{ {1,x,y,x^{2} ,xy,y^{2} ,x^{3} ,x^{2} y,xy^{2} ,y^{3} } \right\},\,\,m = 10.$$

The matrix **P** has a size $N \times m$ and represents the collected values of (5) as

6 $${\mathbf{P}} = \left[ {\begin{array}{*{20}c} {p_{1} \left( {{\mathbf{x}}_{1} } \right)} & \cdots & {p_{m} \left( {{\mathbf{x}}_{1} } \right)} \\ \vdots & \ddots & \vdots \\ {p_{1} \left( {{\mathbf{x}}_{N} } \right)} & \cdots & {p_{m} \left( {{\mathbf{x}}_{N} } \right)} \\ \end{array} } \right],$$

and **r**(**x**) in Eq. (2) has the form of

7 $${\mathbf{r}}\left( {\mathbf{x}} \right) = \left[ {\gamma \left( {{\mathbf{x}},{\mathbf{x}}_{1} } \right) \gamma \left( {{\mathbf{x}},{\mathbf{x}}_{2} } \right) \ldots \gamma \left( {{\mathbf{x}},{\mathbf{x}}_{N} } \right)} \right]^{T} ,$$

where $\gamma \left( {{\mathbf{x}},{\mathbf{x}}_{j} } \right)$ is the correlation between any pair of nodes located at **x** and ${\mathbf{x}}_{j} ,$ and it belongs to the covariance of the field value *u*(**x**), i.e. $\gamma \left( {{\mathbf{x}}_{i} ,{\mathbf{x}}_{j} } \right) = cov\left[ {{\mathbf{u}}_{i} ,{\mathbf{u}}_{j} } \right].$ The correlation matrix ${\mathbf{R}}\left[ {\gamma \left( {{\mathbf{x}}_{i} ,{\mathbf{x}}_{j} } \right)} \right]_{N \times N}$ is given by

8 $${\mathbf{R}} = \left[ {\begin{array}{*{20}c} {\gamma \left( {{\mathbf{x}}_{1} ,{\mathbf{x}}_{1} } \right)} & \cdots & {\gamma \left( {{\mathbf{x}}_{1} ,{\mathbf{x}}_{N} } \right)} \\ \vdots & \ddots & \vdots \\ {\gamma \left( {{\mathbf{x}}_{N} ,{\mathbf{x}}_{1} } \right)} & \cdots & {\gamma \left( {{\mathbf{x}}_{N} ,{\mathbf{x}}_{N} } \right)} \\ \end{array} } \right].$$

Many different correlation functions can be used for the correlation matrix. Gaussian function with a correlation parameter $\theta$ is often used to best fit the model due to its simplicity.

9 $$\gamma \left( {{\mathbf{x}}_{i} ,{\mathbf{x}}_{j} } \right) = e^{{ - \theta r_{ij}^{2} }} ,$$

where $r_{ij} = \left\| {{\mathbf{x}}_{i} - {\mathbf{x}}_{j} } \right\|$ and $\theta > 0$ is a parameter controlling the shape of the correlation function.

Let $C^{{k_{1} }} \left(\Omega \right)$ be the space of $k_{1}$th continuously differentiable functions on $\Omega .$ If $\gamma \left( {{\mathbf{x}},{\mathbf{x}}_{i} } \right) \in C^{{k_{1} }} \left(\Omega \right)$ and $p_{j} \left( {\mathbf{x}} \right) \in C^{{k_{2} }} \left(\Omega \right)$ where $i = 1,2, \ldots ,N$ and $j = 1,2, \ldots ,m$ then $\phi_{i} \left( {\mathbf{x}} \right) \in C^{k} \left(\Omega \right)$ with $k = { \hbox{min} }\left( {k_{1} ,k_{2} } \right).$ The partial derivatives of the shape function ${\varvec{\Phi}}\left( {\mathbf{x}} \right)$ with respect to ${\mathbf{x}}_{i}$ are obtained as

$$\begin{aligned} {\varvec{\Phi}}_{,i} \left( {\mathbf{x}} \right) & = {\mathbf{p}}_{,i}^{T} \left( {\mathbf{x}} \right){\mathbf{A}} + {\mathbf{r}}_{,i}^{T} \left( {\mathbf{x}} \right){\mathbf{B}}, \\ {\varvec{\Phi}}_{,ii} \left( {\mathbf{x}} \right) & = {\mathbf{p}}_{,ii}^{T} \left( {\mathbf{x}} \right){\mathbf{A}} + {\mathbf{r}}_{,ii}^{T} \left( {\mathbf{x}} \right){\mathbf{B}}, \\ \end{aligned}$$

where $\left( \cdot \right)_{,i}$ and $\left( \cdot \right)_{,ii}$ denote the first- and second-order spatial derivatives, respectively.

## Research methodology

In this section we introduce the governing time fractional NSE in Cartesian coordinate system and then the MLPG formulation and numerical implementation including local weak form and discretization techniques are described in detail. Let us now consider the time fractional NSE in two dimensions for an incompressible fluid in the following form:

10 $$\frac{{\partial^{\alpha } u}}{{\partial t^{\alpha } }} + u\frac{\partial u}{\partial x} + v\frac{\partial u}{\partial y} = - \frac{\partial p}{\partial x} + \frac{1}{Re}\left( {\frac{{\partial^{2} u}}{{\partial x^{2} }} + \frac{{\partial^{2} u}}{{\partial y^{2} }}} \right) + f_{x} ,$$

11 $$\frac{{\partial^{\alpha } v}}{{\partial t^{\alpha } }} + u\frac{\partial v}{\partial x} + v\frac{\partial v}{\partial y} = - \frac{\partial p}{\partial y} + \frac{1}{Re}\left( {\frac{{\partial^{2} v}}{{\partial x^{2} }} + \frac{{\partial^{2} v}}{{\partial y^{2} }}} \right) + f_{y} ,$$

12 $$\frac{\partial u}{\partial x} + \frac{\partial v}{\partial y} = 0,$$

where *u*, *v*, and *p* are the velocity components in the x and y directions and pressure, respectively, *Re* represents the Reynolds number, $f_{x}$ and $f_{y}$ stand for externally imposed forces that act throughout the body of fluid along the x and y directions, respectively, $\alpha$ is the parameter indicating the order of the fractional, $0 < \alpha < 1,$ and the time fractional derivative presented herein from Caputo’s viewpoint is defined by

13 $$\frac{{\partial^{\alpha } u\left( {{\mathbf{x}},t} \right)}}{{\partial t^{\alpha } }} = \frac{1}{{\Gamma \left( {1 - \alpha } \right)}}\int\nolimits_{0}^{t} {\frac{{\partial u\left( {{\mathbf{x}},\tau } \right)}}{\partial \tau }\left( {t - \tau } \right)^{ - \alpha } d\tau ,}$$

where ${\Gamma }\left( \cdot \right)$ denotes the gamma function. In the case of ${\upalpha } = 1$, Eqs. (10), (11) and (12) reduce to the classical NSE.

### Spatial discretization

Instead of giving the global weak form, the MLPG method constructs the weak form over local sub-domains, $\Omega _{s} ,$ which is a small region taken for each node in the global domain ${\Omega}.$ The local sub-domains overlap each other and cover the whole global domain. The local sub-domains (support domain) could be of any geometric shape and size, such as open or closed intervals in one dimension, circles or squares in two dimensions and spheres or cubes in three dimensions. For simplicity they are usually taken as a circle. The local weak form for Eqs. (10), (11) and (12) at each ${\mathbf{x}}_{i} \in {\Omega }_{s}^{i}$ can be weighted by test functions and integrated over a local sub-domain. The following equations are obtained:

14 $$\int\nolimits_{{\Omega _{s}^{i} }} {\left( {\frac{{\partial^{\alpha } u}}{{\partial t^{\alpha } }} + u\frac{\partial u}{\partial x} + v\frac{\partial u}{\partial y}} \right)} w\left( {\mathbf{x}} \right)d\Omega = \int\nolimits_{{\Omega _{s}^{i} }} {\left( { - \frac{\partial p}{\partial x} + \frac{1}{Re}\left( {\frac{{\partial^{2} u}}{{\partial x^{2} }} + \frac{{\partial^{2} u}}{{\partial y^{2} }}} \right) + f_{x} \left( {\mathbf{x}} \right)} \right)} w\left( {\mathbf{x}} \right)d\Omega ,$$

15 $$\int\nolimits_{{\Omega _{s}^{i} }} {\left( {\frac{{\partial^{\alpha } v}}{{\partial t^{\alpha } }} + u\frac{\partial v}{\partial x} + v\frac{\partial v}{\partial y}} \right)} w\left( {\mathbf{x}} \right)d\Omega = \int\nolimits_{{\Omega _{s}^{i} }} {\left( { - \frac{\partial p}{\partial y} + \frac{1}{Re}\left( {\frac{{\partial^{2} v}}{{\partial x^{2} }} + \frac{{\partial^{2} v}}{{\partial y^{2} }}} \right) + f_{y} \left( {\mathbf{x}} \right)} \right)} w\left( {\mathbf{x}} \right)d\Omega ,$$

16 $$\int\nolimits_{{\Omega _{s}^{i} }} {\left( {\frac{\partial u}{\partial x} + \frac{\partial v}{\partial y}} \right)} w\left( {\mathbf{x}} \right)d\Omega = 0,$$

where $\Omega _{s}^{i}$ is a local sub-domain associated with the point *i*, i.e. a bounded circle centered at ${\mathbf{x}}_{i}$ of radius $r_{0} ,$ and *w* is a test function.

Substituting the trial function, $u^{h} ,v^{h}$ and $p^{h} ,$ for $u,v$ and *p* into the local weak forms except the nonlinear term gives

17 $$\begin{aligned} & \mathop \sum \limits_{j = 1}^{N} \left( {\int\nolimits_{{\Omega _{s}^{i} }} {\phi_{j} \left( {\mathbf{x}} \right)w\left( {\mathbf{x}} \right)} d\Omega } \right)\frac{{\partial^{\alpha } \hat{u}_{j} \left( t \right)}}{{\partial t^{\alpha } }} + \mathop \sum \limits_{j = 1}^{N} \left[ {\int\nolimits_{{\Omega _{s}^{i} }} {\left( {u\phi_{j,x} \left( {\mathbf{x}} \right) + v\phi_{j,y} \left( {\mathbf{x}} \right) - \frac{1}{Re}\left( {\phi_{j,xx} \left( {\mathbf{x}} \right) + \phi_{j,yy} \left( {\mathbf{x}} \right)} \right)} \right)} w\left( {\mathbf{x}} \right)d\Omega } \right]\hat{u}_{j} \left( t \right) \\ & \quad + \mathop \sum \limits_{j = 1}^{N} \left( {\int\nolimits_{{\Omega _{s}^{i} }} {\phi_{j,x} \left( {\mathbf{x}} \right)w\left( {\mathbf{x}} \right)} d\Omega } \right)\hat{p}_{j} \left( t \right) = \int\nolimits_{{\Omega _{s}^{i} }} {f_{x} \left( {\mathbf{x}} \right)w\left( {\mathbf{x}} \right)} d\Omega , \\ \end{aligned}$$

18 $$\begin{aligned} & \mathop \sum \limits_{j = 1}^{N} \left( {\int\nolimits_{{{{\Omega }}_{s}^{i} }} {\phi_{j} \left( {\mathbf{x}} \right)w\left( {\mathbf{x}} \right)} d\Omega } \right)\frac{{\partial^{\alpha } \hat{v}_{j} \left( t \right)}}{{\partial t^{\alpha } }} + \mathop \sum \limits_{j = 1}^{N} \left[ {\int\nolimits_{{\Omega _{s}^{i} }} {\left( {u\phi_{j,x} \left( {\mathbf{x}} \right) + v\phi_{j,y} \left( {\mathbf{x}} \right) - \frac{1}{Re}\left( {\phi_{j,xx} \left( {\mathbf{x}} \right) + \phi_{j,yy} \left( {\mathbf{x}} \right)} \right)} \right)} w\left( {\mathbf{x}} \right)d\Omega } \right]\hat{v}_{j} \left( t \right) \\ & \quad + \mathop \sum \limits_{j = 1}^{N} \left( {\int\nolimits_{{\Omega _{s}^{i} }} {\phi_{j,y} \left( {\mathbf{x}} \right)w\left( {\mathbf{x}} \right)} d\Omega } \right)\hat{p}_{j} \left( t \right) = \int\nolimits_{{\Omega _{s}^{i} }} {f_{y} \left( {\mathbf{x}} \right)w\left( {\mathbf{x}} \right)} d\Omega , \\ \end{aligned}$$

19 $$\mathop \sum \limits_{j = 1}^{N} \left( {\int\nolimits_{{\Omega _{s}^{i} }} {\phi_{j,x} \left( {\mathbf{x}} \right)w\left( {\mathbf{x}} \right)d\Omega } } \right)\hat{u}_{j} \left( t \right) + \mathop \sum \limits_{j = 1}^{N} \left( {\int\nolimits_{{\Omega _{s}^{i} }} {\phi_{j,y} \left( {\mathbf{x}} \right)w\left( {\mathbf{x}} \right)d\Omega } } \right)\hat{v}_{j} \left( t \right) = 0.$$

In order to avoid the evaluation of any numerical integration in the weak form, the Kronecker delta function is chosen as the test function in each sub-domain. The Kronecker delta function maintains a unit value at the node and gives zero value at all other nodes. For this choice the support domain can be arbitrary. The local integral equations can be simplified to the following expression:

20 $$\begin{aligned} & \mathop \sum \limits_{j = 1}^{N} \phi_{j} \left( {{\mathbf{x}}_{i} } \right)\frac{{\partial^{\alpha } \hat{u}_{j} \left( t \right)}}{{\partial t^{\alpha } }} + \mathop \sum \limits_{j = 1}^{N} \left( {u\left( {{\mathbf{x}}_{i} } \right)\phi_{j,x} \left( {{\mathbf{x}}_{i} } \right) + v\left( {{\mathbf{x}}_{i} } \right)\phi_{j,y} \left( {{\mathbf{x}}_{i} } \right) - \frac{1}{Re}\left( {\phi_{j,xx} \left( {{\mathbf{x}}_{i} } \right) + \phi_{j,yy} \left( {{\mathbf{x}}_{i} } \right)} \right)} \right)\hat{u}_{j} \left( t \right) \\ & \quad + \mathop \sum \limits_{j = 1}^{N} \phi_{j,x} \left( {{\mathbf{x}}_{i} } \right)\hat{p}_{j} \left( t \right) = f_{x} \left( {{\mathbf{x}}_{i} } \right), \\ \end{aligned}$$

21 $$\begin{aligned} & \mathop \sum \limits_{j = 1}^{N} \phi_{j} \left( {{\mathbf{x}}_{i} } \right)\frac{{\partial^{\alpha } \hat{v}_{j} \left( t \right)}}{{\partial t^{\alpha } }} + \mathop \sum \limits_{j = 1}^{N} \left( {u\left( {{\mathbf{x}}_{i} } \right)\phi_{j,x} \left( {{\mathbf{x}}_{i} } \right) + v\left( {{\mathbf{x}}_{i} } \right)\phi_{j,y} \left( {{\mathbf{x}}_{i} } \right) - \frac{1}{Re}\left( {\phi_{j,xx} \left( {{\mathbf{x}}_{i} } \right) + \phi_{j,yy} \left( {{\mathbf{x}}_{i} } \right)} \right)} \right)\hat{v}_{j} \left( t \right) \\ & \quad + \mathop \sum \limits_{j = 1}^{N} \phi_{j,y} \left( {{\mathbf{x}}_{i} } \right)\hat{p}_{j} \left( t \right) = f_{y} \left( {{\mathbf{x}}_{i} } \right), \\ \end{aligned}$$

22 $$\mathop \sum \limits_{j = 1}^{N} \phi_{j,x} \left( {{\mathbf{x}}_{i} } \right)\hat{u}_{j} \left( t \right) + \mathop \sum \limits_{j = 1}^{N} \phi_{j,y} \left( {{\mathbf{x}}_{i} } \right)\hat{v}_{j} \left( t \right) = 0.$$

Equations (20), (21) and (22) can be written in matrix–vector notation as

23 $${\mathbf{A}}\frac{{\partial^{\alpha } {\mathbf{U}}}}{{\partial t^{\alpha } }} + {\mathbf{BU}} = {\mathbf{C}},$$

where $${\mathbf{A}} = \left[ {\begin{array}{*{20}l} \varvec{I} \hfill & 0 \hfill & 0 \hfill \\ 0 \hfill & \varvec{I} \hfill & 0 \hfill \\ 0 \hfill & 0 \hfill & 0 \hfill \\ \end{array} } \right],\quad {\mathbf{B}} = \left[ {\begin{array}{*{20}l} {\varvec{B}_{11} } \hfill & 0 \hfill & {\varvec{B}_{13} } \hfill \\ 0 \hfill & {\varvec{B}_{22} } \hfill & {\varvec{B}_{23} } \hfill \\ {\varvec{B}_{31} } \hfill & {\varvec{B}_{32} } \hfill & 0 \hfill \\ \end{array} } \right],\quad {\mathbf{C}} = \left[ {\begin{array}{*{20}c} {\varvec{F}_{\varvec{x}} } \\ {\varvec{F}_{\varvec{y}} } \\ 0 \\ \end{array} } \right], \quad {\mathbf{U}} = \left[ {\begin{array}{*{20}c} {\hat{\varvec{U}}} \\ {\hat{\varvec{V}}} \\ {\hat{\varvec{P}}} \\ \end{array} } \right],$$

$$\begin{aligned} \varvec{B}_{11} & = \left[ {\varphi_{ij} \left( {u,v} \right)} \right];\quad \varphi_{ij} \left( {u,v} \right) = u\phi_{j,x} \left( {{\mathbf{x}}_{i} } \right) + v\phi_{j,y} \left( {{\mathbf{x}}_{i} } \right) - \frac{1}{Re}\left( {\phi_{j,xx} \left( {{\mathbf{x}}_{i} } \right) + \phi_{j,yy} \left( {{\mathbf{x}}_{i} } \right)} \right), \\ \varvec{B}_{13} & = \left[ {\phi_{j,x} \left( {{\mathbf{x}}_{i} } \right)} \right],\quad \varvec{B}_{22} = \varvec{B}_{11} , \quad \varvec{B}_{23} = \left[ {\phi_{j,y} \left( {{\mathbf{x}}_{i} } \right)} \right],\quad \varvec{B}_{31} = \varvec{B}_{13} , \quad \varvec{B}_{32} = \varvec{B}_{23} , \\ \varvec{F}_{\varvec{x}} & = \left[ {f_{x1} f_{x2} f_{x3} \ldots f_{xN} } \right]^{T}, \quad \varvec{F}_{\varvec{y}} = \left[ {f_{y1} f_{y2} f_{y3} \ldots f_{yN} } \right]^{T} , \\ \hat{\varvec{U}} & = \left[ {\hat{u}_{1} \hat{u}_{2} \hat{u}_{3} \ldots \hat{u}_{N} } \right]^{T} ,\quad \hat{\varvec{V}} = \left[ {\hat{v}_{1} \hat{v}_{2} \hat{v}_{3} \ldots \hat{v}_{N} } \right]^{T}, \quad \hat{\varvec{P}} = \left[ {\hat{p}_{1} \hat{p}_{2} \hat{p}_{3} \ldots \hat{p}_{N} } \right]^{T} , \\ \end{aligned}$$

***I*** is the $N \times N$ identity (unit) matrix and **0** is the $N \times N$ zeros matrix.

### Temporal discretization

For some positive integer $M_{1} ,$ let $\Delta t = \frac{T}{{M_{1} }}$ be the step size of time variable. The nodal points in the time interval $\left[ {0,T} \right]$ are given by $t_{n} = n\Delta t, n = 0,1,2, \ldots ,M_{1} .$ The approximate solutions at the point $u\left( {{\mathbf{x}}_{i} ,t_{n} } \right)$ are abbreviated by $u_{i}^{n} .$ In the current work, we apply a simple quadrature formula to obtain the discrete approximation of the time fractional derivative in Caputo’s sense (Murio 2008),

24 $$\frac{{\partial^{\alpha } {\mathbf{U}}^{n} }}{{\partial t^{\alpha } }} = \sigma_{{\alpha ,\Delta t}} \mathop \sum \limits_{k = 1}^{n} \omega_{k}^{\left( \alpha \right)} \left( {{\mathbf{U}}^{n - k + 1} - {\mathbf{U}}^{n - k} } \right) + O\left( {\Delta t} \right),$$

where $$\omega_{k}^{\left( \alpha \right)} = k^{1 - \alpha } - \left( {k - 1} \right)^{1 - \alpha }$$ and $$\sigma_{{\alpha ,\Delta t}} = \frac{1}{{\Gamma \left( {1 - \alpha } \right)}}\frac{1}{1 - \alpha }\frac{1}{{\Delta t^{\alpha } }}.$$

As shown in Eq. (23), the coefficient matrix **B** is itself a function of unknown *u* and *v*. Traditional iterative technique such as Newton–Raphson method might be applied to treat the nonlinearity, but often that is a very time-consuming process. Alternatively, to balance sufficient accuracy and acceptable computational expense, the linearization method by Taylor series expansion of a function can be adopted to approximate the nonlinear term. Since ${\mathbf{U}}\left( {{\mathbf{x}},t_{n} } \right)$ has the first-order continuous derivative, it follows that

25 $${\mathbf{U}}\left( {{\mathbf{x}},t_{n} } \right) = {\mathbf{U}}\left( {{\mathbf{x}},t_{n - 1} } \right) + O\left( {\Delta t} \right) .$$

Substituting Eq. (24) into Eq. (23), making apply Eq. (25) to the unknown values contained within the matrix **B**, and omitting higher-order terms lead to the $3N \times 3N$ discretized system of linear algebraic equations

26 $$\sigma_{{\alpha ,\Delta t}} {\mathbf{A}}\mathop \sum \limits_{k = 1}^{n} \omega_{k}^{\left( \alpha \right)} \left( {{\mathbf{U}}^{n - k + 1} - {\mathbf{U}}^{n - k} } \right) + {\mathbf{B}}^{n - 1} {\mathbf{U}}^{n} = {\mathbf{C}}^{n} ,$$

or equivalently

27 $$\sigma_{{\alpha ,\Delta t}} {\mathbf{A}}\left( {{\mathbf{U}}^{n} - {\mathbf{U}}^{n - 1} } \right) + {\mathbf{B}}^{n} {\mathbf{U}}^{n} = - \sigma_{{\alpha ,\Delta t}} {\mathbf{A}}\mathop \sum \limits_{k = 2}^{n} \omega_{k}^{\left( \alpha \right)} \left( {{\mathbf{U}}^{n - k + 1} - {\mathbf{U}}^{n - k} } \right) + {\mathbf{C}}^{n} .$$

For $n = 1,$ we get

28 $$\left( {\sigma_{{\alpha ,\Delta t}} {\mathbf{A}} + {\mathbf{B}}^{0} } \right){\mathbf{U}}^{1} = \sigma_{{\alpha ,\Delta t}} {\mathbf{AU}}^{0} + {\mathbf{C}}^{1} ,$$

and for $$n \ge 2,$$

29 $$\left( {\sigma_{{\alpha ,\Delta t}} {\mathbf{A}} + {\mathbf{B}}^{n} } \right){\mathbf{U}}^{n} = \sigma_{{\alpha ,\Delta t}} {\mathbf{A}}\left( {{\mathbf{U}}^{n - 1} - \mathop \sum \limits_{k = 2}^{n} \omega_{k}^{\left( \alpha \right)} \left( {{\mathbf{U}}^{n - k + 1} - {\mathbf{U}}^{n - k} } \right)} \right) + {\mathbf{C}}^{n} .$$

A formula such as this which expresses one unknown value directly in terms of known values is called an explicit formula. This can be easily solved as a linear algebraic system of equations.

## Numerical experiments and results

**Fig. 1:**
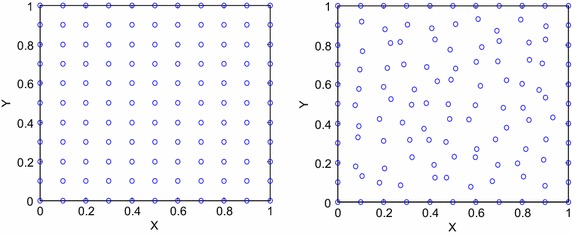
Diagram of regular (*left*) and irregular (*right*) distributions of nodes

In this section, we provide two numerical examples to corroborate the accuracy and efficiency of the proposed method. The results of numerical experiments are compared with analytical solution by performing the root mean square (RMS) error:

30 $$RMS = \sqrt {\frac{1}{N}\mathop \sum \limits_{i = 1}^{N} \left( {U_{i} - u_{i} } \right)^{2} } ,$$

where $U_{i}$ and $u_{i}$ are the analytical and approximate solutions at points ${\mathbf{x}}_{i} ,$ respectively, and *N* is the total number of nodal points. The rate of convergence can be computed approximately by

31 $$\frac{{log\left( {\frac{{E_{1} }}{{E_{2} }}} \right)}}{{log\left( {\frac{{\Delta t_{1} }}{{\Delta t_{2} }}} \right)}},$$

in which $E_{1}$ and $E_{2}$ are errors corresponding to grids with step size of time variable $\Delta t_{1}$ and $\Delta t_{2} ,$ respectively. In the MK procedure, the cubic basis function ${\mathbf{p}}^{T} \left( x \right) = \left\{ {1,x,y,x^{2} ,xy,y^{2} ,x^{3} ,x^{2} y,xy^{2} ,y^{3} } \right\},m = 10,$ is used for all numerical computations because in general a cubic polynomial basis will yield a better result than quadratic and linear basis. The correlation parameter is another factor that has a significant effect on the solution. As a matter of fact, no exact rules can be derived appropriately to determine the optimal value of this parameter. However, it is often suggested to be $\theta = \omega /d_{c}^{2}$ in order to smooth out small features in the data. The numerator $\omega = 0.2$ is used according to the recommendation of Zheng and Dai (2011). The denominator $d_{c}$ is the nodal spacing near the point of interest. If the nodes are uniformly distributed, $d_{c}$ is simply the distance between two neighboring nodes in that the distance between two consecutive nodes in each direction is constant. When nodes are non-uniform, $d_{c}$ is the average distance of the nodes in the support domain. Throughout the experiment, the nodal points are assumed to be both regular (uniform) and irregular (non-uniform) distributions placed in the square domain, i.e. $\Omega = \left[ {a,b} \right] \times \left[ {c,d} \right].$ For some positive integers $M_{2}$ and $M_{3}$, let $\Delta x = \frac{b - a}{{M_{2} }}$ and $\Delta y = \frac{d - c}{{M_{3} }}$ be the step size of space variables in x and y directions, respectively. So we define $x_{i} = a + i\Delta x, i = 0,1,2, \ldots M_{2}$ and $y_{j} = c + j\Delta y, j = 0,1,2, \ldots M_{3}$. Figure 1 shows the diagram of regular and irregular distributions of nodes over the region $\Omega = \left[ {0,1} \right] \times \left[ {0,1} \right] = \left\{ {\left. {\left( {x,y} \right)} \right|0 \le x,y \le 1} \right\}.$

### **Test problem 1**

Consider the time fractional Navier–Stokes equations with the body force problem. For an appropriate polynomial body force, the exact solution of the unsteady-state flow problem with homogeneous boundary conditions becomes

32 $$u\left( {x,y,t} \right) = 2x^{2} y\left( {1 - x} \right)^{2} \left( {1 - y} \right)\left( {1 - 2y} \right)e^{ - t} ,$$

33 $$v\left( {x,y,t} \right) = - 2xy^{2} \left( {1 - x} \right)\left( {1 - 2x} \right)\left( {1 - y} \right)^{2} e^{ - t} ,$$

34 $$p\left( {x,y,t} \right) = (x^{2} - y^{2} )e^{ - t} .$$

**Fig. 2:**
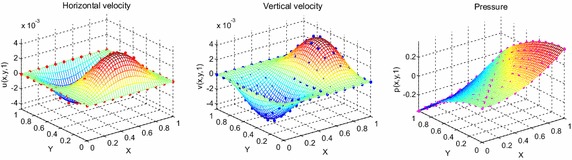
The graphs of approximate and exact solutions after 10 iterations with $\alpha = 0.99,\Delta x =\Delta y = 0.1,\Delta t = 0.1,Re = 100$ for the horizontal (*left*) and vertical (*middle*) components of velocity and pressure (*right*)

**Fig. 3:**
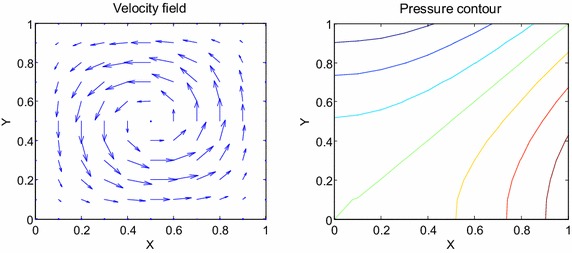
The velocity and pressure fields at $Re = 100$

**Fig. 4:**
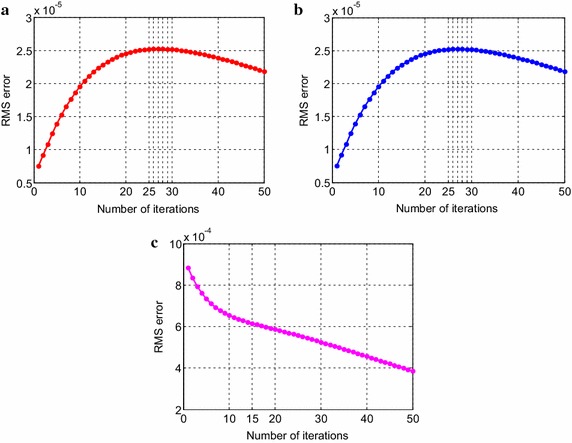
The long-time behavior of RMS errors for the horizontal (**a**) and vertical (**b**) components of velocity and pressure (**c**)

**Fig. 5:**
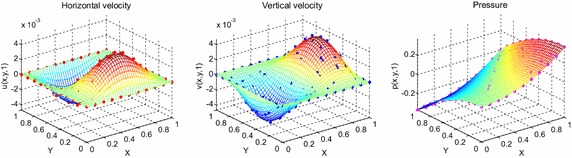
The graphs of approximate and exact solutions after 10 iterations with $\alpha = 0.99,{{\Delta }}x = {{\Delta }}y = 0.1,{{\Delta }}t = 0.1,Re = 100$ for the horizontal (*left*) and vertical (*middle*) components of velocity and pressure (*right*)

**Fig. 6:**
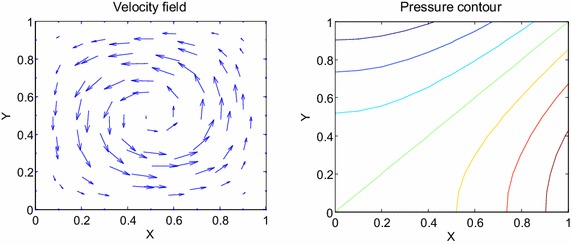
The velocity and pressure fields at $Re = 100$

**Fig. 7:**
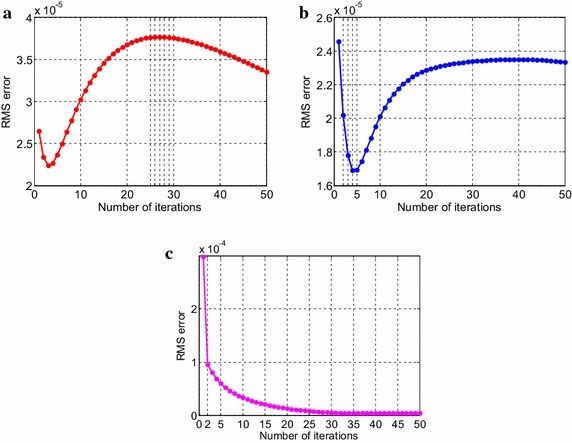
The long-time behavior of RMS errors for the horizontal (**a**) and vertical (**b**) components of velocity and pressure (**c**)

The initial and boundary conditions can be obtained from the exact solution. In this test problem, the nodal distribution is defined on the square domain $\Omega = \left[ {0,1} \right] \times \left[ {0,1} \right].$ In Table 1 we show RMS errors and convergence rate of the velocity and pressure obtained in solving test problem 1 for different values of $\Delta t$ with regular points. Figure 2 shows the graphs of approximate and exact solutions after 10 iterations with $\alpha = 0.99,\Delta x =\Delta y = 0.1,\Delta t = 0.1,Re = 100$ for the horizontal and vertical components of velocity and pressure. The numerical results are shown by dots while the exact solutions are generated by meshes. Also the velocity and pressure fields at $Re = 100$ are plotted in Fig. 3 with $11 \times 11$ points. To observe the behavior of the numerical solutions for long time (50 iterations), the RMS errors are plotted against the number of iterations, indicated in Fig. 4. The applicability of the proposed scheme to irregularly scattered nodes is also given in Table 2, Figs. 5, 6 and 7.

**Table 1:** The *RMS* errors and convergence rate of the velocity and pressure obtained in solving test problem 1 for different values of $\Delta t$ with regular points

$\Delta t$	$\Delta x =\Delta y = 1/10$			Convergence rate		
	*u*	*v*	*p*	*u*	*v*	*p*
1/10	9.9638 × 10^−5^	9.9638 × 10^−5^	6.4379 × 10^−4^	–	–	–
1/12	8.1523 × 10^−5^	8.1523 × 10^−5^	5.9193 × 10^−4^	1.1006	1.1006	0.4606
1/15	6.3501 × 10^−5^	6.3501 × 10^−5^	5.3916 × 10^−4^	1.1196	1.1196	0.4185
1/17	5.5141 × 10^−5^	5.5141 × 10^−5^	5.1400 × 10^−4^	1.1278	1.1278	0.3818
1/20	4.5947 × 10^−5^	4.5947 × 10^−5^	4.8544 × 10^−4^	1.1224	1.1224	0.3518

**Table 2:** The *RMS* errors and convergence rate of the velocity and pressure obtained in solving test problem 1 for different values of $\Delta t$ with random points

$\Delta t$	$\Delta x =\Delta y = 1/10$			Convergence rate		
	*u*	*v*	*p*	*u*	*v*	*p*
1/10	1.1461 × 10^−4^	1.0447 × 10^−4^	4.6520 × 10^−6^	–	–	–
1/12	9.6714 × 10^−5^	8.6530 × 10^−5^	4.1804 × 10^−6^	0.9312	1.0334	0.5863
1/15	7.8888 × 10^−5^	6.8595 × 10^−5^	3.8668 × 10^−6^	0.9130	1.0409	0.3495
1/17	7.0584 × 10^−5^	6.0213 × 10^−5^	3.7879 × 10^−6^	0.8886	1.0413	0.1647
1/20	6.1379 × 10^−5^	5.0900 × 10^−5^	3.7583 × 10^−6^	0.8598	1.0339	0.0483

### **Test problem 2**

The most widely used benchmark tests in incompressible unsteady flow simulation is the Taylor–Green vortex problem. The Taylor–Green vortex is an outstandingly canonical problem in computational fluid dynamics developed to study decaying vortex and turbulent transition. This problem serves as a well-known benchmark case study for testing and validating the reliability of a numerical scheme and is also used to perform significant test on behavior of the velocity and pressure properties. The exact closed form solution for decaying vortex problem is given by

35 $$u\left( {x,y,t} \right) = { \sin }\left( x \right){ \cos }\left( y \right)e^{{\frac{ - 2t}{Re}}} ,$$

36 $$v\left( {x,y,t} \right) = - { \cos }\left( x \right){ \sin }\left( y \right)e^{{\frac{ - 2t}{Re}}} ,$$

37 $$p\left( {x,y,t} \right) = \frac{1}{4}\left( {{ \cos}\,2x + { \cos}\,2y} \right)e^{{\frac{ - 4t}{Re}}} .$$

**Fig. 8:**
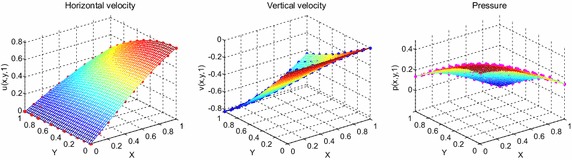
The graphs of approximate and exact solutions after 10 iterations with $\alpha = 0.99,\Delta x =\Delta y = 0.1,\Delta t = 0.1,Re = 100$ for the horizontal (*left*) and vertical (*middle*) components of velocity and pressure (*right*)

**Fig. 9:**
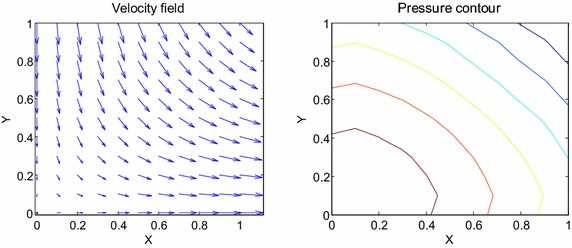
The velocity and pressure fields at $Re = 100$

**Fig. 10:**
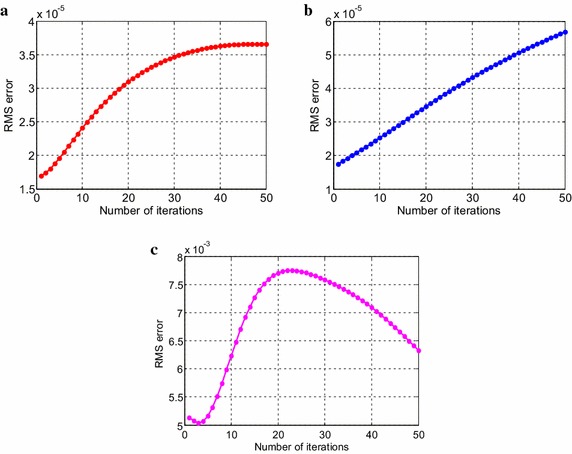
The long-time behavior of RMS errors for the horizontal (**a**) and vertical (**b**) components of velocity and pressure (**c**)

**Fig. 11:**
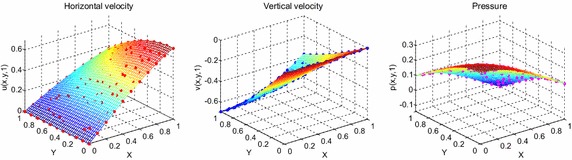
The graphs of approximate and exact solutions after 10 iterations with $\alpha = 0.99,\Delta x =\Delta y = 0.1,\Delta t = 0.1,Re = 10$ for the horizontal (*left*) and vertical (*middle*) components of velocity and pressure (*right*)

**Fig. 12:**
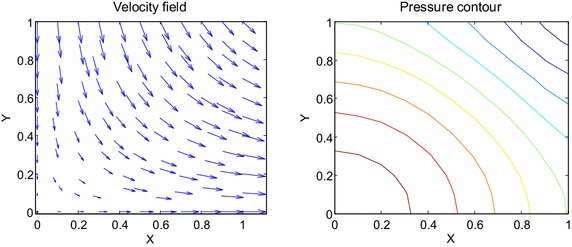
The velocity and pressure fields at $Re = 10$

**Fig. 13:**
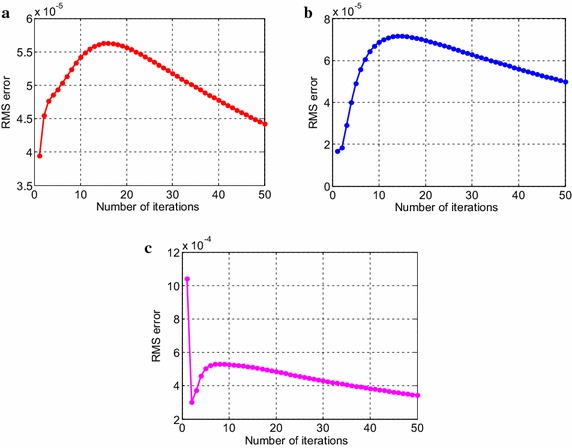
The long-time behavior of RMS errors for the horizontal (**a**) and vertical (**b**) components of velocity and pressure (**c**)

The initial and boundary conditions can be obtained from the exact solution. In test problem 2, we consider a nonlinear model of problem (10)–(12) in the unit square domain $\left( {x,y} \right) \in \left[ {0,1} \right] \times \left[ {0,1} \right],t \in \left[ {0,1} \right].$ Table 3 gives RMS errors and convergence rate of the velocity and pressure obtained in solving test problem 2 for different values of $\Delta t$ with regular points. The graphs of approximate and exact solutions after 10 iterations with $\alpha = 0.99,\Delta x =\Delta y = 0.1,\Delta t = 0.1,Re = 100$ for the horizontal and vertical components of velocity and pressure are depicted in Fig. 8. Under the same illustration as above, the numerical results are shown by dots while the analytical solutions are connected together by meshes. The velocity and pressure fields at $Re = 100$ are also shown in Fig. 9 with $11 \times 11$ points. The long-time behavior of the numerical solutions is displayed in Fig. 10. The distribution with random points is reported in Table 4, Figs. 11, 12 and 13 as well.

**Table 3:** The *RMS* errors and convergence rate of the velocity and pressure obtained in solving test problem 2 for different values of $\Delta t$ with regular points

$\Delta t$	$\Delta x =\Delta y = 1/10$			Convergence rate		
	*u*	*v*	*p*	*u*	*v*	*p*
1/10	2.1744 × 10^−4^	3.0347 × 10^−4^	1.1725 × 10^−2^	–	–	–
1/12	1.7912 × 10^−4^	2.3433 × 10^−4^	1.0747 × 10^−2^	1.0633	1.4181	0.4777
1/15	1.4059 × 10^−4^	1.6391 × 10^−4^	9.7033 × 10^−3^	1.0854	1.6017	0.4578
1/17	1.2243 × 10^−4^	1.3050 × 10^−4^	9.1864 × 10^−3^	1.1050	1.8212	0.4374
1/20	1.0204 × 10^−4^	9.3123 × 10^−5^	8.5828 × 10^−3^	1.1209	2.0764	0.4182

**Table 4:** The *RMS* errors and convergence rate of the velocity and pressure obtained in solving test problem 2 for different values of $\Delta t$ with random points

$\Delta t$	$\Delta x =\Delta y = 1/10$			Convergence rate		
	*u*	*v*	*p*	*u*	*v*	*p*
1/10	3.3935 × 10^−4^	4.2293 × 10^−4^	2.8988 × 10^−3^	–	–	–
1/12	2.7347 × 10^−4^	3.4414 × 10^−4^	2.3473 × 10^−3^	1.1838	1.1307	1.1575
1/15	2.0839 × 10^−4^	2.6582 × 10^−4^	1.8016 × 10^−3^	1.2180	1.1572	1.1857
1/17	1.7815 × 10^−4^	2.2914 × 10^−4^	1.5471 × 10^−3^	1.2526	1.1863	1.2168
1/20	1.4456 × 10^−4^	1.8801 × 10^−4^	1.2629 × 10^−3^	1.2856	1.2173	1.2489

We can verify the temporal numerical accuracy with a fixed and sufficiently small spacing of $\Delta x =\Delta y = 0.1$ and different temporal step sizes $\Delta t.$ As can be seen in Tables 1, 2, 3 and 4, the RMS errors decrease as the number of iteration is increased, that is to say we can get higher accuracy when the temporal step length is decreased. The performance of numerical accuracy is very satisfactory for the present choice of $\Delta x,\Delta y$ and $\Delta t$. In the case of randomly scattered nodes, $\omega = 0.2$ in the empirical formula for the correlation parameter is kept unchanged in the computation. The step size of time variable $\left( {\Delta t} \right)$ is chosen the same as regularly arranged nodes. Comparing the obtained results in Tables 1 and 2 also indicates that the irregular distribution of nodal points leads to better results than the regular distribution for pressure solutions only. The pressure errors for case of irregular nodal distribution are obviously much smaller than those of regular nodal distribution whilst the velocity solutions give slightly different numerical results. What is more, we notice from Fig. 4 that the velocity errors gradually reduce when the process is iterated until reaching to 25th calculation whilst the pressure errors behave like an exponentially decreasing function for the first few time steps and then seem to be reduced linearly, approximately near n = 15. The behavior of the solutions in Fig. 7c is also observed. The errors decrease exponentially after only a few time steps. It evidently dramatically tends to zero. In test problem 2, it is noteworthy that when the uneven nodal points are considered, accurate results are obtained at low Reynolds numbers. At $Re = 100,$ the numerical error is not good enough. Herein, the Reynolds number is selected to be 10. Other Reynolds number values are possible but the error increases and an undesirable result is unavoidable. Presumably this is because the exact solution of the Taylor–Green vortex problem not only decays exponentially with time but also depends on the Reynolds number which has a significant effect on the solution. More investigation is needed. Nevertheless, the results presented through these figures and tables show the performance and validity of the proposed method to approximate the exact solution with high accuracy. All in all the results obtained from both of the test problem 1 and 2 are congruous with what we expected. In special case, when $\alpha \to 1,$ it can be seen that the numerical results depicted in Figs. 2, 5, 8 and 11 tend to the analytical solution of classical NSE. Moreover, it is worth pointing out that the behavior of the solution by fractional model can be observed as the fractional derivative parameter is changed.

## Conclusion

In this paper, we have presented a numerical scheme used to obtain the approximate solution of the time fractional Navier–Stokes equations. The truly meshless local Petrov–Galerkin approach based on a local weak formulation is employed to approximate the solution of field variables. The Kronecker delta function is chosen as the test function in each sub-domain to avoid the evaluation of any numerical integration in weak formulation. We apply the effective moving Kriging interpolation which possesses the delta function property for constructing shape functions at scattered points. A quadrature formula is used to obtain the discrete formulation of the time fractional derivative interpreted in the sense of Caputo. Besides, the nonlinear parts of the time fractional NSE can be treated by a simple linearization method based on Taylor series expansion. The capability and accuracy of the proposed scheme is demonstrated through solving the body force and Taylor–Green vortex problems. Very good agreement between the analytical and numerical results can be found. It is apparent that the present algorithm based on MLPG method can readily be extended to solve the unsteady incompressible Navier–Stokes equations involving non-integer order time derivative and is also found to be a computationally efficient and reliable method to deal with FDE.
